# Patients posture affects clinical outcomes and range of motion after reverse total shoulder arthroplasty: A clinical study

**DOI:** 10.1016/j.jseint.2024.10.002

**Published:** 2024-11-09

**Authors:** Philipp Kriechling, Georgios Neopoulos, Alexander Berger, Philipp Stein, Tobias Götschi, Florian Grubhofer, Karl Wieser

**Affiliations:** Balgrist University Hospital, Orthopaedic Department, University of Zurich, Zurich, Switzerland

**Keywords:** Scapulothoracic orientation, Posture, Shoulder osteoarthritis, Reverse shoulder arthroplasty, Range of motion, Clinical outcome

## Abstract

**Background:**

Movement limitations following implantation of reverse total shoulder arthroplasty (rTSA) have been observed in some patients postoperatively, with implant design and positioning recognized as important influential factors. Recent analyses have identified patient’s posture, measured as scapula internal rotation on computed tomography (CT), as an additional factor influencing the functional outcome after rTSA. However, no clinical study has correlated the preoperatively photo-documented posture to functional outcome. It was the aim of this study to correlate preoperatively photo-documented posture to scapula orientation using CT and analyze the influence on functional outcome following rTSA implantation.

**Methods:**

A prospectively enrolled rTSA database was retrospectively reviewed to include a total of 360 patients with a minimum follow-up of 2 years. Patient’s posture was analyzed using standardized preoperative photo and video documentation. The posture was defined following the classification system of Moroder et al as type A (upright posture, retracted scapulae), type B (intermediate), and type C (kyphotic posture with protracted scapulae). In addition, CT data were used to measure scapula position (internal rotation). Correlation analyses between them were conducted. Postoperative range of motion (ROM) and clinical outcomes (absolute Constant-Murley Score and relative Constant-Murley Score) were compared between the different posture types.

**Results:**

According to the photo-documented posture types, the patients were divided into posture types A (n = 59), B (n = 253) and C (n = 48). Average absolute Constant-Murley Score differed significantly among the groups (69 ± 16 vs. 69 ± 14 vs. 64 ± 16, *P* < .05) favoring patients with posture types A and B over type C. In terms of ROM, flexion, abduction, and internal rotation significantly differed among the groups. Types A and B exhibited better flexion and abduction (flexion 124 ± 26° and 123 ± 23° vs. 113 ± 25°, abduction 140 ± 34° and 137 ± 30° vs. 128 ± 34°). Patients with posture type A demonstrated superior internal rotation (CS points: 5.9 ± 2.9 vs. 5.0 ± 2.7 vs. 4.4 ± 2.8, *P* < .05). External rotation was better for type A compared to type C (A: 33 ± 17° vs. B: 30 ± 16° vs. C: 28 ± 18°). Correlation analysis of posture classification using photo documentation and CT scan showed poor reliability (r = 0.35).

**Conclusion:**

Patients with clinical posture types A and B exhibited improved ROM values compared to type C postures. Clinical outcome scores were also notably superior in types A and B. However, the measurement of scapula internal rotation on supine CT does not reliably correlate with photo documentation of patient’s posture. Preoperatively, patient’s posture should be considered in rTSA planning because of the potential influence on ROM and clinical outcomes.

Reverse total shoulder arthroplasty (rTSA) is gaining popularity for the treatment of shoulder pathologies, such as rotator cuff tear arthropathy, massive irreparable rotator cuff tear, osteoarthritis, rheumatoid arthritis, and fracture, as well as a revision option with increasing implantation rates due to the aging population.^5^^,^^9^^,^^19^ In rTSA following Grammont’s principles,^11^ the rotational center of the glenohumeral joint shifts medially and inferiorly and is stabilized through the use of a more constrained design. This design alters the native anatomy, impacting the biomechanics of the arm elevation as well as the external and internal rotation.^4^^,^^11^

Limitations in movement, particularly internal and external rotation, have been observed in many patients following rTSA.^7^^,^^15^^,^^20^^,^^30^^,^^31^ Various implant designs and positioning parameters, such as glenoid component size, lateral offset, inferior overhang, the neck-shaft angle, and torsion of the humeral component have been identified to determine the achievable range of motion (ROM).^28^

Recently analyzed, scapular orientation was highly variable in the population.^22^ Especially, internal rotation and protraction might be influenced by the thoracic kyphosis and could show increased values in elderly patients. Moroder et al introduced a three-type classification system to define the patient’s posture as type A (upright), type B (intermediate), and type C (kyphotic) based on the scapular internal rotation measured on a supine computed tomography (CT) of the shoulder.^22^ According to their study, changes in orientation of the scapula, especially in protraction and internal rotation could potentially influence the opposition of the humeral component to the glenosphere. This, in turn, could affect the impingement free ROM.^24^ In a modeling study, irrespective of the rTSA implant configuration, they demonstrated inferior ROM for patients with posture type C (thoracic kyphosis, strong scapula internal rotation and protraction) compared to those with types A and B.^24^ In a recently published clinical study, patients classified as posture type C, based on scapular internal rotation measured on a supine CT scan, displayed a lower abduction and flexion, along with lower clinical scores and increased pain following rTSA implantation compared to those with posture type A and B.^23^ However, the study design did not allow a correlation of CT measurement for scapula orientation to the preoperative clinical posture of the patients.

To the best of our knowledge, no study has analyzed the functional outcome in association with patient’s photo-documented posture. Further, no study has correlated posture analysis using photo documentation with evaluation on CT.

Therefore, it was the study aim to fill this gap in the literature investigating the relationship between the patient’s photo-documented posture and clinical outcome and to further validate correlation between photo documentation and CT measurement of patient’s posture.

It was hypothesized that there could be a correlation between the clinical posture and scapular internal rotation measured on the supine CT scan and that both posture and scapula orientation significantly influence the postoperative functional outcome after rTSA.

## Material and methods

### Study design and inclusion and exclusion criteria

This study received approval from the cantonal ethical committee. The research was exclusively conducted at the author’s institution. A prospectively enrolled rTSA database was retrospectively reviewed. The inclusion criteria were as follows: patients underwent primary RTSA implantation, availability of preoperative shoulder CT and photo/video documentation for evaluation of the patient’s posture, patient’s approval for study inclusion, age over 18 years, and a minimum follow-up of 2 years to evaluate clinical outcome including ROM. The exclusion criteria were as follows: patients with incomplete follow-up; those who declined the use of their records for the study; cases involving revision rTSA; and patients with documented complications during follow-up that could potentially affect the ROM including instability, periprosthetic fractures, infection, nerve injuries, loosening of the components, or any other reason for revision.^19^ The rTSA database that started in September 2005 was then retrospectively analyzed backwards from 2021 in accordance with the power analysis (see statistical section below).

### Surgical technique

All patients were treated using the Anatomical Shoulder Inverse/Reverse prosthesis (Zimmer Biomet, Warsaw, IN, USA), through the commonly described deltopectoral approach. The surgical procedures were executed in a standardized manner, consistent with the previously described protocol.^18^ The humeral component (onlay, 155° neck shaft angle) was inserted, aiming for a retroversion not exceeding 0° to −20° in all patients. Additional cementation was decided on intraoperatively, depending on bone quality and press-fit stem fixation. The glenoid baseplate was implanted in a neutral version and neutral to slightly inferior inclination not exceeding 10°. Whenever possible, a transosseous refixation of the subscapularis tendon using No. 2 FiberWire (Arthrex, Naples, FL, USA) was also carried out in neutral or slight external rotation of the arm to avoid stiffness for external rotation. Postoperatively, all patients were required to wear a sling for a duration of 4-6 weeks. Subsequently, physiotherapy measures with passive and active-assisted mobilization of the shoulder were implemented.

### Clinical and radiographic evaluation

Demographic data included in the analysis were age, gender, operated side, smoking and alcohol status, the American Society of Anesthesiologists score and body mass index. All patients underwent routine preoperative examination and were assessed at 6 weeks, 18 weeks, and at 1 and 2 years. ROM as well as pain assessment were documented preoperatively and at every follow-up. The absolute Constant-Murley Score and the relative Constant-Murley Score (CSa/CSr)^8^ as well as the Subjective Shoulder Value^10^ were determined preoperatively, at 1 and 2 years postoperatively. Internal rotation was measured using the CS classification that gave 10 points (best score) for reaching the interscapular region (T7), 8 points for the inferior tip of the scapula (T12), 6 points for the waist (L3), 4 points for the lumbosacral junction, 2 points the buttock 2 points, and 0 points the lateral thigh.

**Figure 1:**
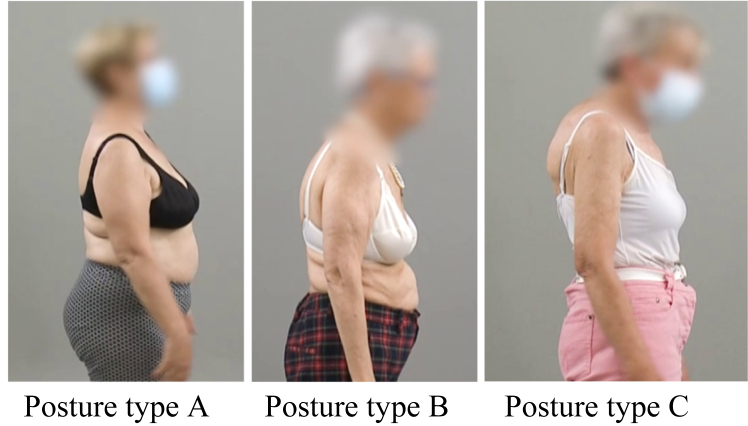
Preoperative clinical (photo-documented) posture types following the classification of Moroder et al: type A (upright posture, retracted scapulae), type B (intermediate), and type C (kyphotic posture with protracted scapulae).

Preoperatively, photo and video documentation of patient’s shoulder ROM and posture were performed in a standing position. Using the video footage, posture type A (upright posture, retracted scapulae), type B (intermediate), and type C (kyphotic posture with protracted scapulae) were evaluated as previously described.^22^ All postures were determined for every patient independently by two senior orthopedic residents with special interest in shoulder surgery (GN, AB). In cases of differences between the readers, a consensus was reached (Fig. 1).

**Figure 2:**
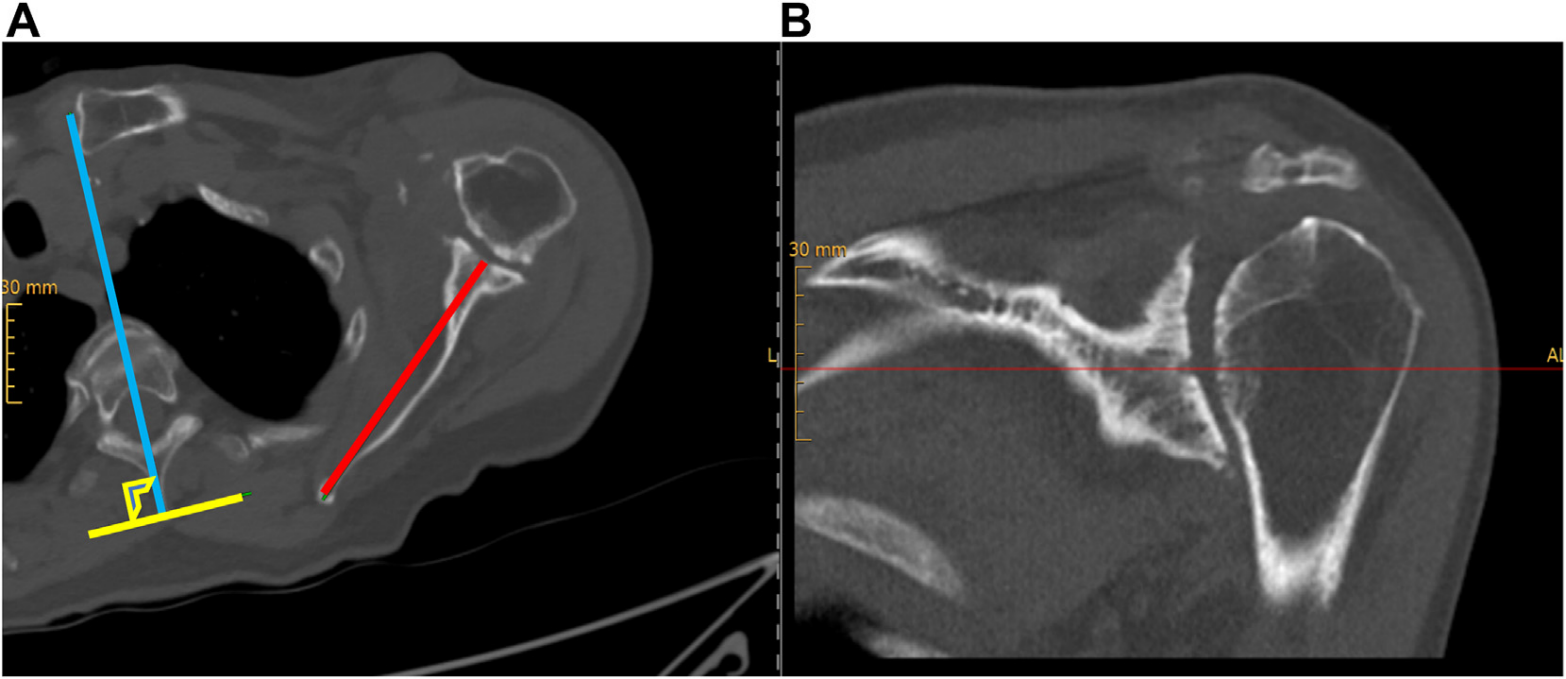
A computed tomography of a left shoulder in the axial (**A**) and coronal plane (**B**). The deepest point of the glenoid in both axial and coronal plane was defined. The *blue line* determines the parallel line to the spinal process, defining the axis of the vertebra. The *yellow line* shows the perpendicular line to the vertebral axis. The *red line* represents the line from the deepest point of the glenoid to the medial root of the scapula spine in the axial plane. The internal rotation of the scapula was then measured as the angle between the *yellow line* and the *red line*.

Further, routine preoperative evaluation included x-rays in three standardized planes (true anteroposterior, lateral scapula, and axial view), supplemented by additional imaging through supine CT for planning purposes. The measurement of the internal rotation of the scapula was performed by the same two authors (GN, AB) using the preoperative supine CT. To determine the scapula orientation and to prevent any anatomical bias, three bony landmarks were utilized. These landmarks included: the deepest point of the glenoid concavity in both the coronal and axial plane, the inferior scapula angle and the medial root of the scapular spine, following the methodology of Park et al and Moroder et al.^22^^,^^26^ The internal rotation was then measured as the angle between the perpendicular line to the sagittal vertebral axis and the line from the deepest point of the glenoid to the medial root of the scapula spine in the axial plane (Fig. 2).

For correlation between photo documentation and CT measurement, internal rotation measurements were defined as follows: type A as internal rotation equal or less than 36°, type B as internal rotation between 36° and 47°, and type C as internal rotation equal or larger than 47° according to Moroder et al.^22^^,^^24^

### Statistical analysis

The collected data and measurements were initially anonymized and transferred to the REDCap system (Vanderbilt University, Nashville, TN, USA)^13^^,^^14^ hosted at Balgrist University Hospital.

The statistical analysis was performed using RStudio (Version 2023.12.0 Build 369; Posit, Boston, MA, USA). Distribution of the data was tested using the Shapiro-Wilk test and visual inspection. Correlation between the scapular internal rotation and the preoperative clinical posture of the patients was tested using the Spearman rank correlation test (r). Interrater reliability between the two authors was analyzed using Cohen’s kappa and intraclass correlation coefficients as appropriate. Comparison of the scapular internal rotation, the postoperative ROM and the clinical outcomes among the groups was performed using Kruskal-Wallis rank sum test for three-groups followed by the Wilcoxon rank sum test to identify potential significant differences between the groups. Prior to patient inclusion, a comprehensive power analysis was carried out aiming for a statistical power of 0.8. The power analysis also included the fact that the different groups would be of different size when analyzing a subsequent series of patients. Accordingly, the determined sample size for this study was set at 360 patients. The level of significance was set to *P* < .05.

## Results

### Demographics and posture type

**Figure 3:**
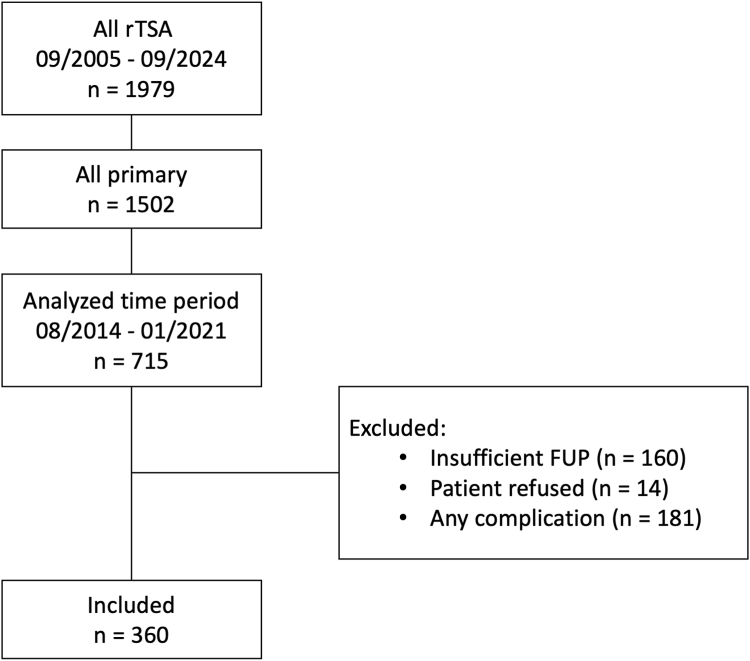
Flowchart displaying the selection process. *FUP*, follow-up; *n*, number; rTSA, reverse total shoulder arthroplasty.

As determined by the power analysis, the study included 360 rTSAs with implantation between August 2014 and January 2021 at an average age of 71 ± 9 years at a mean follow-up of 24.5 ± 1.4 months (flowchart Fig. 3). Among these, 219 patients (61%) were female, and the average body mass index was 27.7 ± 5.7 kg/m^2^. The preoperative video footage of the patients in the lateral standing position revealed that 59 patients (16.4%) exhibited posture type A, 253 patients (70.3%) posture type B and 48 patients (13.3%) type C. Basic demographic data were comparable between the groups and are summarized in Table I.

**Table I:** Demographics.

Characteristic	Overall, N = 360∗	A, N = 59∗	B, N = 253∗	C, N = 48∗
Age at surgery	71 ± 9	69 ± 9	71 ± 9	74 ± 8
Female	219 (61%)	44 (75%)	144 (57%)	31 (65%)
Right	221 (61%)	37 (63%)	156 (62%)	28 (58%)
BMI	27.7 ± 5.7	25.8 ± 4.7	28.1 ± 5.4	28.1 ± 7.5
Alcohol				
No	208 (58%)	34 (58%)	148 (59%)	26 (54%)
Rarely	73 (20%)	15 (25%)	51 (20%)	7 (15%)
Regular	56 (16%)	7 (12%)	39 (16%)	10 (21%)
Abuse	10 (2.8%)	3 (5.1%)	4 (1.6%)	3 (6.3%)
Unknown	13 (3.6%)	0 (0%)	11 (4.3%)	2 (4.2%)
Nicotin				
Yes	48 (13%)	11 (19%)	28 (11%)	9 (19%)
Stopped	50 (14%)	6 (10%)	36 (14%)	8 (17%)
Never	255 (71%)	42 (71%)	184 (73%)	29 (60%)
Unknown	7 (1.9%)	0 (0%)	5 (2.0%)	2 (4.2%)
ASA score				
1	16 (4.4%)	6 (10%)	10 (4.0%)	0 (0%)
2	213 (59%)	44 (75%)	143 (57%)	26 (54%)
3	130 (36%)	9 (15%)	99 (39%)	22 (46%)
4	1 (0.3%)	0 (0%)	1 (0.4%)	0 (0%)
Indication				
AVN	4 (1.1%)	0 (0%)	1 (0.4%)	3 (6.3%)
Crystal arthropathy	1 (0.3%)	0 (0%)	1 (0.4%)	0 (0%)
CTA	16 (4.4%)	3 (5.1%)	7 (2.8%)	6 (13%)
Fracture acute	2 (0.6%)	1 (1.7%)	0 (0%)	1 (2.1%)
Fracture conversion plate	1 (0.3%)	0 (0%)	0 (0%)	1 (2.1%)
Instability	5 (1.4%)	1 (1.7%)	3 (1.2%)	1 (2.1%)
OA	87 (24%)	11 (19%)	65 (26%)	11 (23%)
RCT	137 (38%)	24 (41%)	99 (39%)	14 (29%)
RCT + OA	107 (30%)	19 (32%)	77 (30%)	11 (23%)

*BMI*, body mass index; *ASA*, American Society of Anesthesiologists; *AVN*, avascular necrosis; *CTA*, cuff tear arthropathy; *OA*, osteoarthritis; *RCT*, rotator cuff tears; *SD*, standard deviation.

∗ Mean ± SD; n (%).

### Clinical outcomes

The average CSa differed significantly among the posture types: 69 ± 16 in patients with posture type A, 69 ± 14 in patients with posture B, and 64 ± 16 in patients with posture type C (*P* < .05). Subgroup analysis revealed a significantly better CSa in patients with posture type A and B compared to patients with type C (*P* < .05). However, no significant difference between posture types A and B was found.

A CSr (%) subgroup analysis underlined the significant differences in favor of patients with posture type A and B over type C (*P* < .05) but no difference between posture types A and B (*P* = .8).

Regarding postoperative abduction strength, the averages were 3.1 ± 1.9 kg for patients with posture type A, 3.6 ± 2 kg for patients with posture type B, and 2.7 ± 1.9 kg for patients with posture type C with a significant difference between the groups (*P* < .05).

**Figure 4:**
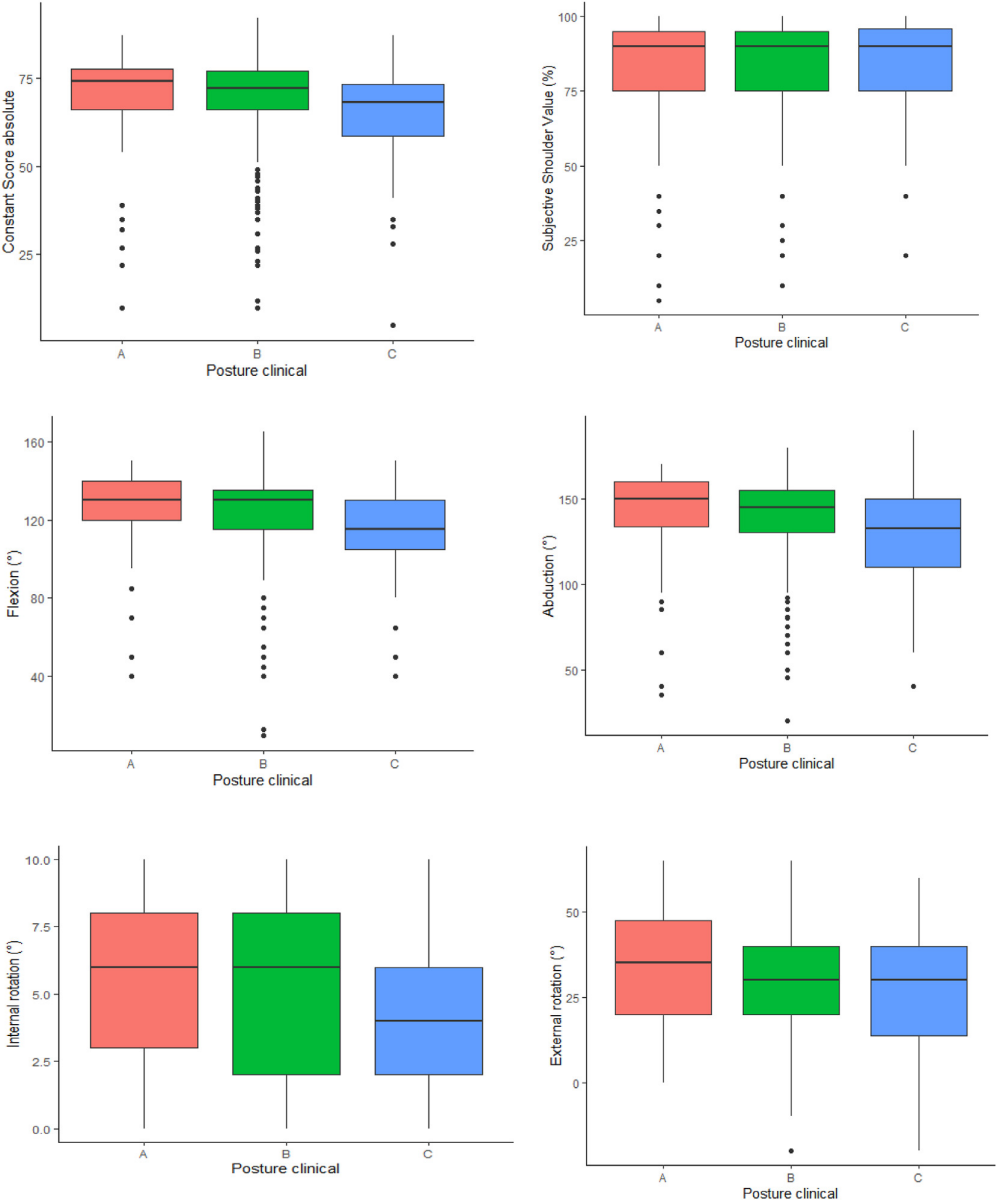
Outcome boxplots visualizing the different parameters (absolute Constant Score, subjective shoulder value, flexion, abduction, internal rotation, and external rotation) to compare the different clinical (photo-documented) posture types.

Detailed information is provided in Table II, Supplemental Tables I-III and Fig. 4.

**Table II:** Comparison among three groups with different posture types evaluated on photo documentation.

Characteristic	A, N = 59∗	B, N = 253∗	C, N = 48∗	*P* value†
IR on CT (°)	34.6 ± 3.5§‖	37.3 ± 5.2‡‖	40.8 ± 4.9‡§	<.001
CSa	69 ± 16‖	69 ± 14‖	64 ± 16‡§	.014
CSr (%)	81 ± 18‖	82 ± 16‖	78 ± 17‡§	.075
SSV (%)	81 ± 23	82 ± 19	83 ± 19	.8
Flexion (°)	124 ± 26‖	123 ± 23‖	113 ± 25‡§	.003
Abduction (°)	140 ± 34‖	137 ± 30‖	128 ± 34‡§	.012
IR	5.86 ± 2.94§‖	5.00 ± 2.68‡	4.38 ± 2.79‡	.020
ER (°)	33 ± 17	30 ± 16	28 ± 18	.3
Strength (kg)	3.08 ± 1.87	3.55 ± 1.95‖	2.72 ± 1.88§	.012

*CSa*, absolute Constant-Murley Score; *CSr*, relative Constant-Murley Score; *ER*, external rotation; *IR*, internal rotation; *SSV*, Subjective Shoulder Value; *SD*, standard deviation.

∗ Mean ± SD.

† Kruskal-Wallis rank sum test.

‡ Shows significant difference compared to group A using Wilcoxon-Rank sum test.

§ Shows significant difference compared to group B using Wilcoxon-Rank sum test.

‖ Shows significant difference compared to group C using Wilcoxon-Rank sum test.

### Range of motion

The flexion, abduction, and internal rotation differed significantly among the groups.

Subgroup analysis revealed that patients with posture type A and B had a significantly better flexion and abduction compared to patients with posture type C (*P* < .05). Internal rotation was much better in posture type A over types B and C (*P* < .05). However, the difference in favor of type B over type C was not statistically different (*P* = .14). Type A patients showed the best ability to externally rotate and patients in group C the worst without a statistically relevant difference. Table II, Supplemental Tables I-III and Fig. 3 provide detailed information of all data.

### Correlation of clinical posture and radiographic evaluation

**Figure 5:**
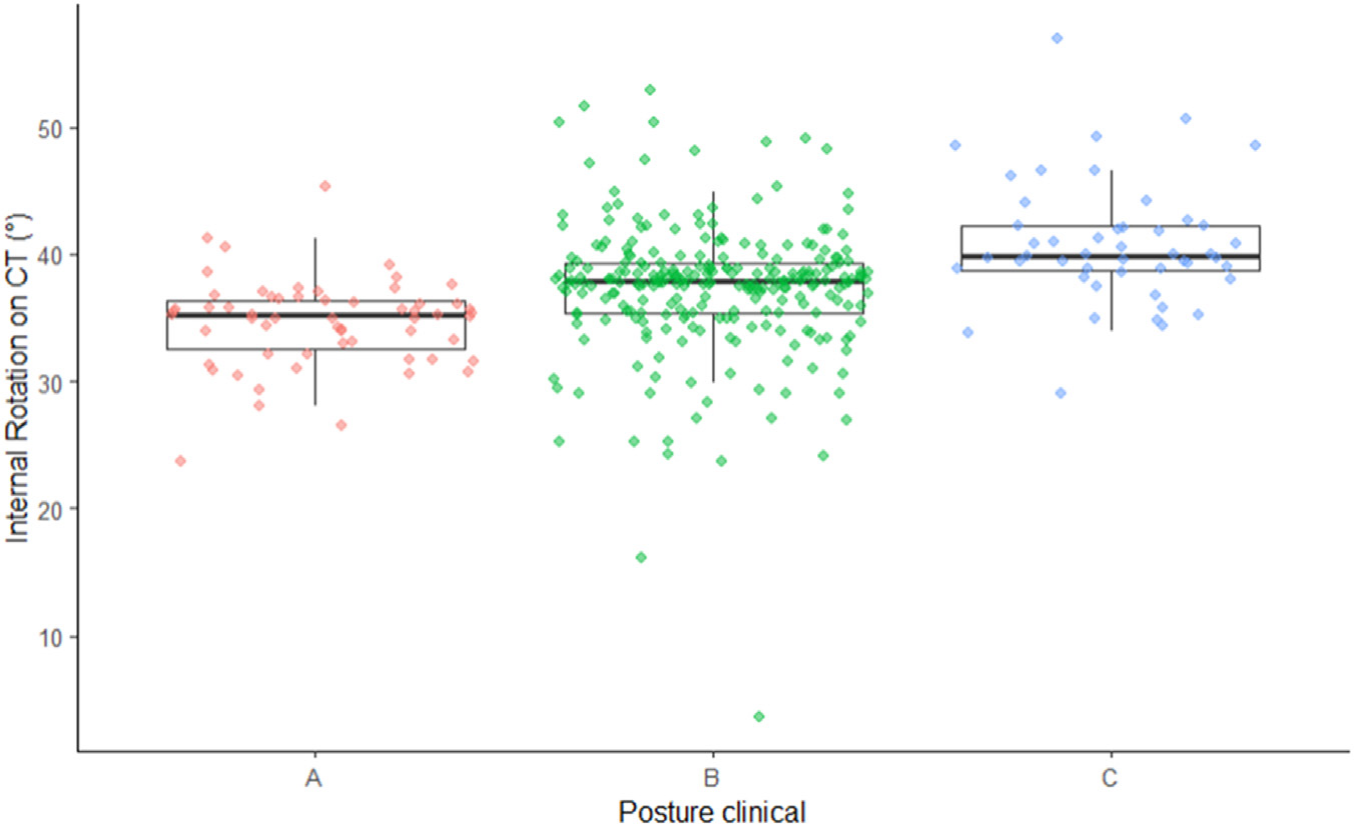
Correlation of clinical posture and internal rotation of the scapula on CT. *CT*, computed tomography.

The correlation of the standing photo-documented posture evaluation differed significantly from the measurement of the scapula orientation (internal rotation in °) using supine CTs (Table III). Clinical classification using photo documentation revealed 59 (16%), 253 (70%), and 48 (13%) in groups A, B, and C, respectively. CT analysis resulted in 121 (34%), 221 (61%), and 18 (5%) in groups A, B, and C, respectively. Comparison of clinical posture type with orientation of the scapula measured as internal orientation showed a weak association using the Spearman-Rank correlation test (r = 0.35) (Fig. 5). Evaluation of clinical outcome and ROM using the CT measurements for posture classification is provided in Supplemental Tables IV-VII.

**Table III:** Shows the correlation of posture type as defined by clinical assessment (photo documentation) and via measurement of scapula orientation on CT.

	Type A	Type B	Type C
Classified on photos			
N	59 (16%)	253 (70%)	48 (13%)
Angled measured (CT) (mean ± SD (min; max))	35 ± 3 (24; 45)	37 ± 5 (3; 53)	4 1 ± 5 (29; 57)
Classified on CT			
N	121 (34%)	221 (61%)	18 (5%)
Angle measured (CT) (mean ± SD (min; max))	32 ± 4 (4; 36)	39 ± 2 (36; 45)	49 ± 3 (46; 57)

*CT*, computed tomography; *SD*, standard deviation.

### Interrater reliability for measurement

Interrater reliability for photo-documented posture (clinical evaluation) revealed a high correlation between the two readers of 0.865 (Cohen’s kappa). Angle measurement on CTs also showed a good correlation of 0.779 (95% confidence interval, 0.735-0.816) measured as intraclass correlation coefficients.

## Discussion

The most important findings of this study were as follows: (1) the correlation between patient’s posture assessed via photo documentation (clinical assessment) and measurement of scapula orientation on CT was poor (Correlation r = 0.35) and (2) patient’s posture type C documented on preoperative photos/videos was associated with worse clinical outcome, especially ROM.

Moroder et al^22^ proposed a classification system of patient’s posture showing a consistency between scapula orientation and thoracic kyphosis. Further, they analyzed the simulated impact on virtually implanted rTSA with different combinations of components, designs and position.^24^ This led to a large dataset showing significantly worse simulated ROM depending on certain factors but largely depending on the scapula orientation of the patient. One of the main results was that a patient with type C posture showed the worst clinical outcome but would markedly benefit from a more retroverted stem, less neck-shaft-angle (f.i., 135° more favorable than 155°) and a larger and inferiorized glenosphere.^24^^,^^29^

A pivotal point influencing the impingement-free ROM is humeral component retrotorsion. A large number of biomechanical studies, all of them not including patients’ posture, have yielded varying results regarding optimal humeral retrotorsion and impingement-free internal and external rotation.^2^^,^^3^^,^^12^^,^^16^^,^^17^^,^^25^^,^^31^^,^^32^ Other authors published that less retrotorsion of 0°-20° would improve impingement free ROM and would result in better outcomes for daily activities of living.^12^^,^^32^ Contrary to that, others found the optimal range to be between 20° and 40° of retrotorsion for achieving an impingement-free internal and external rotation.^31^ So far, no consensus on the angle of humeral retrotorsion could be reached.^1^^,^^6^^,^^27^ However, again, none of these studies considered patient posture as an influential factor.

If an individual choice of implant position with respect to patients’ posture could eventually mitigate the decreased ROM observed in patients with a posture type C remains hypothetical and was not analyzed by this study or any other clinical study so far.

Very recently, Moroder et al also analyzed patient’s scapula orientation in a clinical setting. Despite not analyzing preoperative photo-documented posture, they found a correlation with inferior clinical outcomes and decreased flexion and abduction associated with patients with posture type C compared to those with types A and B.^23^ However, the patients were categorized into posture types according to the scapula internal rotation measured from supine CT scans.

Our study could confirm these results partially but found a weak correlation between the patient’s clinical posture, assessed from standing lateral preoperative photo and scapular orientation measured as internal rotation on supine CT scans.

In our study, the actual internal rotation of the scapula on CT (type A internal rotation < 36°, type B 36°-47°, type C > 47°) by itself did not reveal significant differences in clinical outcome and ROM (see Supplemental Tables IV-VII). One plausible explanation could be the supine position of the patients during CT scans which might alter the angle of scapular internal rotation.

However, our data showed a clear correlation between patient’s photo-documented posture and clinical outcome, indicating that the clinical posture of the patient could help anticipate the clinical outcomes and the postoperative ROM. Patients with posture types A and B demonstrated significantly better abduction and flexion compared to those with posture type C, which also confirms the findings of Moroder et al.^23^ Furthermore, in contrast with the other available clinical study by Moroder et al, patients with type A also had superior internal rotation compared to those with type B and C. In terms of external rotation, although the values were better in patients with posture type A, the difference between the other groups was not statistically significant.

Our evaluation using CT measurements revealed more patients with posture type A and type B than type C. One explanation might be that patient’s posture and scapula internal rotation are not consistently associated to each other or that patient’s posture is dynamic and influenced by lying supine on the flat table during CT scan.

Moroder et al^22^ initially found a good correlation between the correction angle and the scapula orientation of R = 0.71 and moderate to poor correlation to scapula protraction of R = 0.39. Patient’s kyphosis itself showed a poor correlation of R = 0.27 to the scapula internal rotation and of R = 0.57 to the scapula protraction which might explain our deviating findings for clinically documented patient’s posture and CT measurement of internal rotation. However, that only suggests that analysis of the scapula orientation using standard CT might be inferior compared to clinical assessment. This was already shown by Matsumura et al^21^ comparing CTs in a standing position to CTs in supine position with some change of the scapula position. Those results can be discussed in either direction as they seem not be deviating too much from a clinical perspective (Supine: 32° ± 65° internal rotation, 12° ± 5° anterior tilt, 16° ± 4° upward rotation; standing: 30° ± 6° internal rotation, 8° ± 5°anterior tilt, 10° ± 5° upward rotation).^21^ Nevertheless, maybe the combination of them all leads to a completely different situation.^21^ Another explanation might be that the cutoffs of lower 36° for type A, between 36° and 47° for type B and more than 47° for type C could be refined because we also found a progressive internal rotation of the scapula but to a smaller degree as proposed.

### Limitations

The study is constrained by the inherent limitations of a retrospective data analysis in a prospectively enrolled cohort. Notably, the CT scans were conducted with patients lying supine on the table, while assessments of patient’s posture relied on photos and videos captured in the standing upright position. A comprehensive three-dimensional analysis of the scapula, which would offer a more precise measurement of the scapula internal rotation, was not conducted. This introduces the possibility of bias in the correlation of the scapular internal rotation and patient’s posture, particularly since performing a CT scan of the patient’s shoulder in the upright position is currently not feasible on a standardized basis, but might give more insight if performed in a controlled study set-up.

Furthermore, other factors that could potentially influence the postoperative ROM, such as positioning parameters (eg, size of the glenoid component, lateralization, inferior overhang), humeral component retrotorsion, surgical indications as well as the status of the rotator cuff tendons were not taken into account in the analysis of the postoperative ROM.

## Conclusion

Scapulothoracic orientation, particularly scapular internal rotation measured on the supine CT scan, does not reliably correlate with the clinically documented patient’s posture. Following rTSA implantation, patients with clinical posture types A (upright posture) and B (intermediate) demonstrate significantly improved abduction and flexion compared to those with posture type C (kyphotic posture). Internal rotation is notably better in patients with posture type A, whereas external rotation displays better values, without reaching statistical significance. Clinical outcomes (CSa, CSr) were also notably superior in types A and B compared to type C. Performing a CT scan with the patients in a standing position may be necessary to evaluate the patient’s scapular orientation and posture, which plays an important role influencing ROM and clinical outcomes after rTSA and should be considered when planning the rTSA.

## Disclaimers:

Funding: No funding was disclosed by the authors.

Conflicts of interest: Karl Wieser is a consultant for Arthrex Inc. and Zurimed AG. All the other authors, their immediate families, and any research foundation with which they are affiliated have not received any financial payments or other benefits from any commercial entity related to the subject of this article.

## Appendix A Supplementary data

Supplemental Tables I-VII
