# Supplementary material for: Patients posture affects clinical outcomes and range of motion after reverse total shoulder arthroplasty: A clinical study
Source: JSES Int. 2024 Nov 9;9(2):445–52. doi: 10.1016/j.jseint.2024.10.002 (PMC11962612; doi:10.1016/j.jseint.2024.10.002)
Supplement: Supplemental Tables I-VII [file mmc1.docx]

Supplemental material

# Supplemental table 1: Subgroup analysis between photo documented group A and B

| **Characteristic** | **A**, N = 59^1^ | **B**, N = 253^1^ | **p-value**^2^ |
| --- | --- | --- | --- |
| IR on CT (°) | 34.6 ± 3.5 | 37.3 ± 5.2 | <0.001 |
| CSa | 69 ± 16 | 69 ± 14 | 0.5 |
| CSr (%) | 81 ± 18 | 82 ± 16 | 0.8 |
| SSV (%) | 81 ± 23 | 82 ± 19 | 0.7 |
| Flexion (°) | 124 ± 26 | 123 ± 23 | 0.2 |
| Abduction (°) | 140 ± 34 | 137 ± 30 | 0.11 |
| IR | 5.86 ± 2.94 | 5.00 ± 2.68 | 0.032 |
| ER(°) | 33 ± 17 | 30 ± 16 | 0.3 |
| Strength (kg) | 3.08 ± 1.87 | 3.55 ± 1.95 | 0.11 |
| ^1^Mean ± SD | | | |
| ^2^Wilcoxon rank sum test  CSa: absolute Constant-Murley Score, CSr: relative Constant-Murley Score, ER: external rotation, IR: internal rotation, SSV – Subjective Shoulder Value | | | |

# Supplemental table 2: Subgroup analysis between photo documented group A and C

| **Characteristic** | **A**, N = 59^1^ | **C**, N = 48^1^ | **p-value**^2^ |
| --- | --- | --- | --- |
| IR on CT (°) | 34.6 ± 3.5 | 40.8 ± 4.9 | <0.001 |
| CSa | 69 ± 16 | 64 ± 16 | 0.010 |
| CSr (%) | 81 ± 18 | 78 ± 17 | 0.049 |
| SSV (%) | 81 ± 23 | 83 ± 19 | 0.8 |
| Flexion (°) | 124 ± 26 | 113 ± 25 | 0.003 |
| Abduction (°) | 140 ± 34 | 128 ± 34 | 0.012 |
| IR | 5.86 ± 2.94 | 4.38 ± 2.79 | 0.009 |
| ER(°) | 33 ± 17 | 28 ± 18 | 0.2 |
| Strength (kg) | 3.08 ± 1.87 | 2.72 ± 1.88 | 0.3 |
| ^1^Mean ± SD | | | |
| ^2^Wilcoxon rank sum test  CSa: absolute Constant-Murley Score, CSr: relative Constant-Murley Score, ER: external rotation, IR: internal rotation, SSV – Subjective Shoulder Value | | | |

# Supplemental table 3: Subgroup analysis between photo documented group A and C

| **Characteristic** | **B**, N = 253^1^ | **C**, N = 48^1^ | **p-value**^2^ |
| --- | --- | --- | --- |
| IR on CT (°) | 37.3 ± 5.2 | 40.8 ± 4.9 | <0.001 |
| CSa | 69 ± 14 | 64 ± 16 | 0.006 |
| CSr (%) | 82 ± 16 | 78 ± 17 | 0.030 |
| SSV (%) | 82 ± 19 | 83 ± 19 | 0.5 |
| Flexion (°) | 123 ± 23 | 113 ± 25 | 0.002 |
| Abduction (°) | 137 ± 30 | 128 ± 34 | 0.016 |
| IR | 5.00 ± 2.68 | 4.38 ± 2.79 | 0.14 |
| ER(°) | 30 ± 16 | 28 ± 18 | 0.4 |
| Strength (kg) | 3.55 ± 1.95 | 2.72 ± 1.88 | 0.006 |
| ^1^Mean ± SD | | | |
| ^2^Wilcoxon rank sum test  CSa: absolute Constant-Murley Score, CSr: relative Constant-Murley Score, ER: external rotation, IR: internal rotation, SSV – Subjective Shoulder Value | | | |

Supplemental tables analyzing outcome according to internal rotation on CT

# Supplemental table 4: Clinical outcome in accordance to scapula internal rotation measured

on CT

| **Characteristic** | **CT A**, N = 121^1^ | **CT B**, N = 221^1^ | **CT C**, N = 18^1^ | **p-value**^2^ |
| --- | --- | --- | --- | --- |
| IR on CT (°) | 32.3 ± 4.4 | 39.1 ± 2.0 | 49.3 ± 2.6 | <0.001 |
| CSa | 68 ± 15 | 69 ± 14 | 66 ± 12 | 0.4 |
| CSr (%) | 81 ± 17 | 82 ± 16 | 80 ± 15 | 0.7 |
| SSV (%) | 81 ± 22 | 82 ± 19 | 83 ± 17 | 0.9 |
| Flexion (°) | 121 ± 24 | 122 ± 24 | 118 ± 18 | 0.3 |
| Abduction (°) | 137 ± 31 | 137 ± 32 | 134 ± 29 | 0.7 |
| IR | 5.14 ± 2.85 | 5.05 ± 2.73 | 4.67 ± 2.66 | 0.8 |
| ER(°) | 30 ± 17 | 30 ± 16 | 34 ± 18 | 0.5 |
| Strength (kg) | 3.33 ± 1.89 | 3.43 ± 2.01 | 2.74 ± 1.47 | 0.4 |
| ^1^Mean ± SD | | | | |
| ^2^Kruskal-Wallis rank sum test  CSa: absolute Constant-Murley Score, CSr: relative Constant-Murley Score, ER: external rotation, IR: internal rotation, SSV – Subjective Shoulder Value | | | | |

# Supplemental table 5: Subgroup analysis between CT group A and CT group B

| **Characteristic** | **CT A**, N = 121^1^ | **CT B**, N = 221^1^ | **p-value**^2^ |
| --- | --- | --- | --- |
| IR on CT (°) | 32.3 ± 4.4 | 39.1 ± 2.0 | <0.001 |
| CSa | 68 ± 15 | 69 ± 14 | 0.7 |
| CSr (%) | 81 ± 17 | 82 ± 16 | 0.7 |
| SSV (%) | 81 ± 22 | 82 ± 19 | 0.7 |
| Flexion (°) | 121 ± 24 | 122 ± 24 | 0.7 |
| Abduction (°) | 137 ± 31 | 137 ± 32 | >0.9 |
| IR | 5.14 ± 2.85 | 5.05 ± 2.73 | 0.8 |
| ER(°) | 30 ± 17 | 30 ± 16 | 0.6 |
| Strength (kg) | 3.33 ± 1.89 | 3.43 ± 2.01 | 0.8 |
| ^1^Mean ± SD | | | |
| ^2^Wilcoxon rank sum test  CSa: absolute Constant-Murley Score, CSr: relative Constant-Murley Score, ER: external rotation, IR: internal rotation, SSV – Subjective Shoulder Value | | | |

# Supplemental table 6: Subgroup analysis between CT group A and CT group C

S

| **Characteristic** | **CT A**, N = 121^1^ | **CT C**, N = 18^1^ | **p-value**^2^ |
| --- | --- | --- | --- |
| IR on CT (°) | 32 ± 4 | 49 ± 3 | <0.001 |
| CSa | 68 ± 15 | 66 ± 12 | 0.3 |
| CSr (%) | 81 ± 17 | 80 ± 15 | 0.5 |
| SSV (%) | 81 ± 22 | 83 ± 17 | >0.9 |
| Flexion (°) | 121 ± 24 | 118 ± 18 | 0.2 |
| Abduction (°) | 137 ± 31 | 134 ± 29 | 0.4 |
| IR | 5.14 ± 2.85 | 4.67 ± 2.66 | 0.5 |
| ER(°) | 30 ± 17 | 34 ± 18 | 0.4 |
| Strength (kg) | 3.33 ± 1.89 | 2.74 ± 1.47 | 0.2 |
| ^1^Mean ± SD | | | |
| ^2^Wilcoxon rank sum test  CSa: absolute Constant-Murley Score, CSr: relative Constant-Murley Score, ER: external rotation, IR: internal rotation, SSV – Subjective Shoulder Value | | | |

# Supplemental table 7: Subgroup analysis between CT group B and CT group C

| **Characteristic** | **CT B**, N = 221^1^ | **CT C**, N = 18^1^ | **p-value**^2^ |
| --- | --- | --- | --- |
| IR on CT (°) | 39.1 ± 2.0 | 49.3 ± 2.6 | <0.001 |
| CSa | 69 ± 14 | 66 ± 12 | 0.2 |
| CSr (%) | 82 ± 16 | 80 ± 15 | 0.4 |
| SSV (%) | 82 ± 19 | 83 ± 17 | 0.8 |
| Flexion (°) | 122 ± 24 | 118 ± 18 | 0.15 |
| Abduction (°) | 137 ± 32 | 134 ± 29 | 0.4 |
| IR | 5.05 ± 2.73 | 4.67 ± 2.66 | 0.6 |
| ER(°) | 30 ± 16 | 34 ± 18 | 0.2 |
| Strength (kg) | 3.43 ± 2.01 | 2.74 ± 1.47 | 0.2 |
| ^1^Mean ± SD | | | |
| ^2^Wilcoxon rank sum test  CSa: absolute Constant-Murley Score, CSr: relative Constant-Murley Score, ER: external rotation, IR: internal rotation, SSV – Subjective Shoulder Value | | | |
